# Heterologous expression of a fully active *Azotobacter vinelandii* nitrogenase Fe protein in *Escherichia coli*

**DOI:** 10.1128/mbio.02572-23

**Published:** 2023-11-01

**Authors:** Joseph B. Solomon, Yiling A. Liu, Kamil Górecki, Robert Quechol, Chi Chung Lee, Andrew J. Jasniewski, Yilin Hu, Markus W. Ribbe

**Affiliations:** 1 Department of Molecular Biology and Biochemistry, University of California, Irvine, California, USA; 2 Department of Chemistry, University of California, Irvine, California, USA,; The University of Arizona, Tucson, Arizona, USA

**Keywords:** nitrogenase, Fe protein, NifH, heterologous expression, assembly

## Abstract

The functional versatility of the Fe protein, the reductase component of nitrogenase, makes it an appealing target for heterologous expression, which could facilitate future biotechnological adaptations of nitrogenase-based production of valuable chemical commodities. Yet, the heterologous synthesis of a fully active Fe protein of *Azotobacter vinelandii* (*Av*NifH) in *Escherichia coli* has proven to be a challenging task. Here, we report the successful synthesis of a fully active *Av*NifH protein upon co-expression of this protein with *Av*IscS/U and *Av*NifM in *E. coli*. Our metal, activity, electron paramagnetic resonance, and X-ray absorption spectroscopy/extended X-ray absorption fine structure (EXAFS) data demonstrate that the heterologously expressed *Av*NifH protein has a high [Fe_4_S_4_] cluster content and is fully functional in nitrogenase catalysis and assembly. Moreover, our phylogenetic analyses and structural predictions suggest that *Av*NifM could serve as a chaperone and assist the maturation of a cluster-replete *Av*NifH protein. Given the crucial importance of the Fe protein for the functionality of nitrogenase, this work establishes an effective framework for developing a heterologous expression system of the complete, two-component nitrogenase system; additionally, it provides a useful tool for further exploring the intricate biosynthetic mechanism of this structurally unique and functionally important metalloenzyme.

**IMPORTANCE:**

The heterologous expression of a fully active *Azotobacter vinelandii* Fe protein (AvNifH) has never been accomplished. Given the functional importance of this protein in nitrogenase catalysis and assembly, the successful expression of AvNifH in *Escherichia coli* as reported herein supplies a key element for the further development of heterologous expression systems that explore the catalytic versatility of the Fe protein, either on its own or as a key component of nitrogenase, for nitrogenase-based biotechnological applications in the future. Moreover, the “clean” genetic background of the heterologous expression host allows for an unambiguous assessment of the effect of certain nif-encoded protein factors, such as AvNifM described in this work, in the maturation of AvNifH, highlighting the utility of this heterologous expression system in further advancing our understanding of the complex biosynthetic mechanism of nitrogenase.

## INTRODUCTION

**Fig 1:**
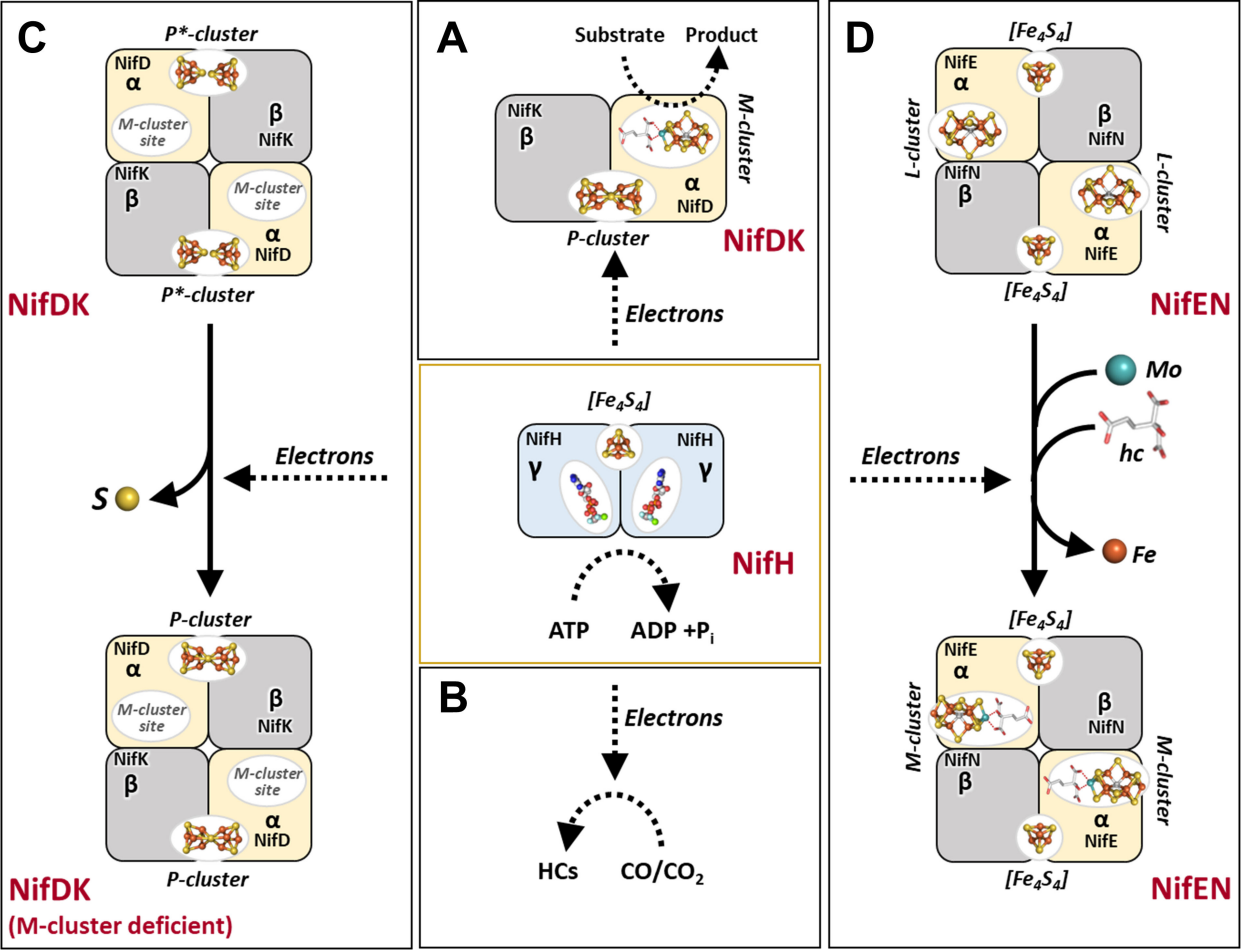
Roles of the Fe protein in substrate reduction and metallocluster assembly. Shown are the functions of Fe protein (NifH) as (A) an electron donor for its catalytic partner, MoFe protein (NifDK), in nitrogenase catalysis; (B) an independent reductase for the reduction of CO or CO_2_ into hydrocarbons (HCs); (C) an insertase of Mo/homocitrate (hc) for the maturation of an L-cluster (i.e., M-cluster precursor [Fe_8_S_9_C]) into a fully assembled M-cluster ([(*R*-homocitrate)MoFe_7_S_9_C]) on a biosynthetic scaffold, NifEN; and (D) a maturase for the reductive coupling of a P*-cluster (i.e., P-cluster precursor; an [Fe_4_S_4_]-like cluster pair) into a P-cluster ([Fe_8_S_7_]). All functions of the Fe protein require an electron source (A–D) and, while its functions in nitrogenase catalysis (A) and biosynthesis (C and D) also rely on ATP hydrolysis, its function as an independent reductase (B) can be accomplished with or without MgATP. For the purpose of simplicity, only one αβ-dimer is shown for the tetrameric NifDK in (A). The atoms of the metalloclusters are colored as follows: Fe, orange; S, yellow; Mo, cyan; C, light gray; Mg, green; O, red; Al, dark gray; F, light blue; and P, dark orange.

The Fe protein of nitrogenase is a versatile FeS enzyme that is crucial for small-molecule activation under ambient conditions (1–3). Recognized mainly for its role as the reductase component of nitrogenase, the Fe protein of the Mo-dependent nitrogenase from the diazotrophic bacterium *Azotobacter vinelandii* (designated *Av*NifH) is an ~60 kDa homodimer that features a subunit-bridging [Fe_4_S_4_] cluster and an MgATP-binding site within each subunit (1–7). In the nitrogenase reaction, *Av*NifH works in concert with its catalytic partner, MoFe protein (designated *Av*NifDK), to enable ATP-dependent electron transfer from the [Fe_4_S_4_] cluster of *Av*NifH, through the [Fe_8_S_7_] P-cluster, to the [(R-homocitrate)-MoFe_7_S_9_C] M-cluster of *Av*NifDK, where the reduction of various substrates, such as N_2_, H^+^, C_2_H_2_, and CO, occurs under ambient conditions (Fig. 1A) (3, 5, 8–13). In addition to its essential role as an electron donor to its catalytic partner during substrate reduction, *Av*NifH can act as an independent reductase and catalyze the *in vivo* and *in vitro* transformation of CO_2_ to CO at its [Fe_4_S_4_] center in the presence or absence of ATP (Fig. 1B) (14, 15). Extending its functions from catalysis to biosynthesis, *Av*NifH also plays a key role in the maturation of both P- and M-clusters, the two unique, high-nuclearity metal centers within *Av*NifDK that are central to the reactivity of nitrogenase. Regarding P-cluster assembly, *Av*NifH facilitates the coupling of a [Fe_4_S_4_] cluster pair into a P-cluster at the α/β-subunit interface of *Av*NifDK (Fig. 1C) (16–19). In the context of M-cluster assembly, *Av*NifH assists in inserting Mo and homocitrate (20–22) into an [Fe_8_S_9_C] precursor on *Av*NifEN (23, 24), a biosynthetic protein with strong sequence and structural resemblance to *Av*NifDK (25), to generate a mature M-cluster that is subsequently delivered to the cofactor-binding site within *Av*NifDK (Fig. 1D) (3, 26). In both biosynthetic processes, *Av*NifH likely interacts with its assembly partners in a way that mirrors its interaction with its catalytic partner during substrate turnover, functioning as an ATP-dependent reductase to enable the maturation of the complex P- and M-clusters of *Av*NifDK.

The ability of the Fe protein to act as a multifunctional reductase hinges on the capability of its [Fe_4_S_4_] cluster to undergo facile redox changes. In the case of *Av*NifH, its [Fe_4_S_4_] cluster is known to adopt three different oxidation states: the oxidized state ([Fe_4_S_4_]^2+^), reduced state ([Fe_4_S_4_]^1+^), and the super-reduced, all-ferrous state ([Fe_4_S_4_]^0^) (1, 2, 11, 27). While ferredoxins or flavodoxins are likely to function as the physiological electron donors for *Av*NifH under *in vivo* conditions, chemical reductants are typically used as the artificial electron donors for *Av*NifH under *in vitro* conditions (3–5, 11). An example of the chemical reductants is Eu^II^-DTPA (with *E*_1/2_ = −1.14 V at pH 8) (2, 28, 29), which is employed with or without ATP in the *in vitro* reduction of CO_2_ by *Av*NifH. In this process, the [Fe_4_S_4_] cluster of *Av*NifH exists in the all-ferrous ([Fe_4_S_4_]^0^) state, which can undergo a reversible one- or two-electron redox transition of the cluster to the reduced ([Fe_4_S_4_]^1+^) or oxidized ([Fe_4_S_4_]^2+^) state to enable substrate reduction (14, 30). Another commonly used chemical reductant is dithionite (e.g., with *E*_1/2_ = −0.47 V at 2 mM dithionite at pH 8), which is used along with MgATP to facilitate the *in vitro* reduction of N_2_ by the complete nitrogenase enzyme. In this case, the [Fe_4_S_4_] cluster of *Av*NifH is maintained in the reduced ([Fe_4_S_4_]^1+^) state, which can undergo a reversible one-electron redox reaction to the oxidized ([Fe_4_S_4_]^2+^) state to enable ATP-dependent electron transfer to the active-site cofactor of *Av*NifDK for substrate reduction (11). It should be noted that the binding of MgATP induces a conformational change of *Av*NifH concomitant with a decrease of the midpoint potentials of its associated [Fe_4_S_4_] cluster by 140 mV (to ca*.* −430 mV) (4), which facilitates the protein-protein interaction between *Av*NifH and *Av*NifDK while promoting the inter-protein transfer of electrons from the former to the latter (1–6, 11). Interestingly, while *Av*NifH interacts with its catalytic and assembly partners in an analogous, ATP-dependent manner, a recent study using a [Fe_4_Se_4_] substituted *Av*NifH as a probe has revealed a difference in the redox requirements of these events, with the catalytic reactions by nitrogenase necessitating a lower reduction potential than the assembly processes of its metalloclusters (29).

The functional versatility of Fe protein makes it an appealing target for heterologous expression in a genetically amenable host like *Escherichia coli*, which could prove useful for developing nitrogenase-based biotechnological adaptations for the production of valuable chemical commodities in the future. Yet, the heterologous synthesis of a fully functional Fe protein in *E. coli* has thus far met with mixed results. Previous efforts have led to the heterologous expression and partial purification of the *Klebsiella pneumoniae* NifH protein from *E. coli*, as well as the successful purification and characterization of the methanogen NifH protein expressed in the same heterologous host (31–33). In the case of *A. vinelandii*, however, the heterologous expression of *Av*NifH with a high FeS content has not been demonstrated in a foreign host like *E. coli*. The challenge posed by this task has motivated the consideration of two key parameters for the successful heterologous synthesis of this unique metalloenzyme. One of them is the [Fe_4_S_4_] cluster of *Av*NifH, the synthesis of which is carried out by NifS/U in the native *A. vinelandii* host, with NifS acting as a pyridoxal 5′-phosphate-dependent cysteine desulfurase enzyme that provides sulfur to the scaffold protein NifU for the sequential synthesis of [Fe_2_S_2_] and [Fe_4_S_4_] clusters. For the heterologous expression of many FeS proteins in *E. coli*, IsuS/U—the homologs of NifS/U—have been successfully used to supplement the endogenous FeS assembly pathways in the expression host and bolster the FeS contents of the heterologously expressed proteins (34–37). The other parameter is the protein scaffold of *Av*NifH, the proper folding of which requires NifM, a protein with a partial sequence similarity to known peptidyl-prolyl isomerases and therefore, presumed to enable a *cis-trans* isomerization of the conserved prolines in *Av*NifH to facilitate its assembly. Given these considerations, it can be rationalized that co-expression of *Av*NIfH with *Av*IscS/U and *Av*NifM could result in the heterologous synthesis of a functional, FeS-cluster replete form of *Av*NifH in *E. coli*.

Here, we report the successful synthesis of a fully active *Av*NifH protein upon co-expression of this protein with *Av*IscS/U and *Av*NifM in *E. coli*. Our metal, activity, electron paramagnetic resonance (EPR), and XAS (X-ray absorption spectroscopy)/EXAFS data demonstrate a high occupancy of [Fe_4_S_4_] clusters and a full spectrum of catalytic and biosynthetic activities of the heterologously expressed *Av*NifH protein. Moreover, our phylogenetic analyses and structural predictions lead to the proposal that *Av*NifM functions as a chaperone that assists the maturation of a cluster-replete *Av*NifH protein. As such, this work provides a useful platform for developing an expression system for the heterologous synthesis of a complete nitrogenase while supplying an effective tool for further investigating the biosynthetic mechanism of this important metalloenzyme.

## RESULTS AND DISCUSSION

**Fig 2:**
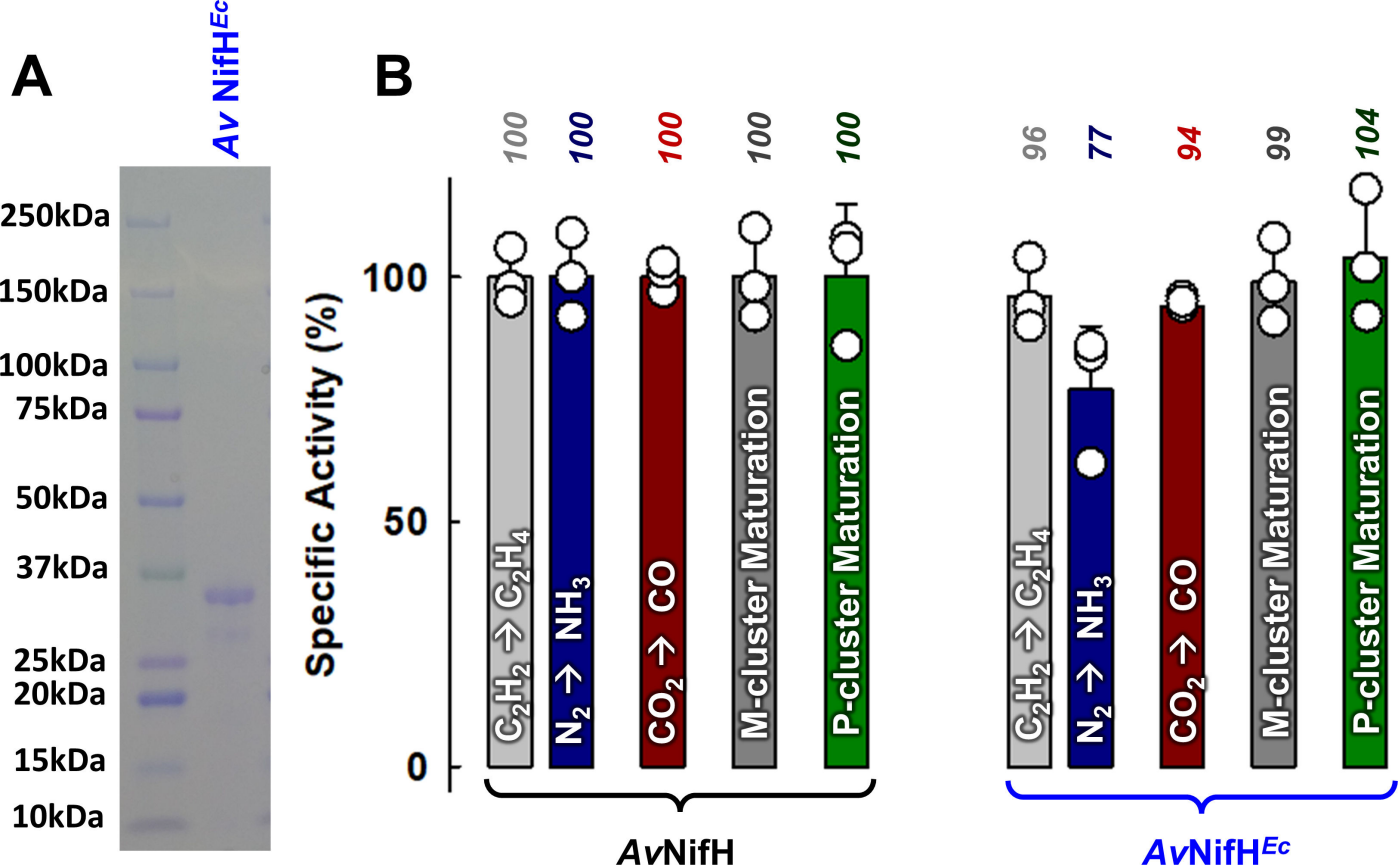
Biochemical and catalytic analyses of *Av*NifH*^Ec^*. (A) SDS-PAGE of the heterologously expressed *Av*NifH*^Ec^*. (B) Specific activities of C_2_H_2_-reduction (to C_2_H_4_; light gray), N_2_-reduction (to NH_3_; dark blue), CO_2_-reduction (to CO; dark red), M-cluster maturation (dark gray), and P-cluster maturation (dark green) by *Av*NifH*^Ec^* as compared to those by its native *Av*NifH counterpart. Shown above the bars are the activities expressed in percentages, with the activities of *Av*NifH set as 100% and the activities of *Av*NifH*^Ec^* calculated relative to those of *Av*NifH (see Table S1 for details). The specific activities are normalized based on the Fe contents of *Av*NifH*^Ec^* (3.3 ± 0.2 mol Fe/mol protein) and *Av*NifH (3.9 ± 0.3 mol Fe/mol protein). The data points shown in panel (B) represent biological replicates (*n* = 3) and are expressed as mean ± standard deviation.

Co-expression of *Av*NifH with *Av*IscS/U and *Av*NifM in *E. coli* strain BL21(DE3) resulted in a brown, soluble protein (designated *Av*NifH*^Ec^*) that was purified at a yield of ∼100 mg protein per 35 g wet cells. A homodimer comprising subunits of ~30 kDa (Fig. 2A), the heterologously expressed *Av*NifH*^Ec^* shows a metal content of 3.3 ± 0.2 mol Fe/mol protein, ~85% of the metal content of 3.9 ± 0.3 mol Fe/mol protein for the *Av*NifH protein isolated from the native *A. vinelandii* host (Table S1). Given the presence of one [Fe_4_S_4_] cluster in this protein, the Fe content of *Av*NifH*^Ec^* suggests an occupancy of >80% of the single [Fe_4_S_4_] cluster-binding site at its dimeric interface (see Fig. 1). Such a cluster assignment of *Av*NifH*^Ec^* also aligns well with its respective activities in CO_2_-reduction (as an independent enzyme), C_2_H_2_- and N_2_-reduction (with *Av*NifDK as its catalytic partner), and P- and M-cluster maturation (with precursor-containing *Av*NifDK and *Av*NifEN as its respective biosynthetic partners), which range from 77% to 104% of those of its native *Av*NifH counterpart (Fig. 2B; Table S1).

**Fig 3:**
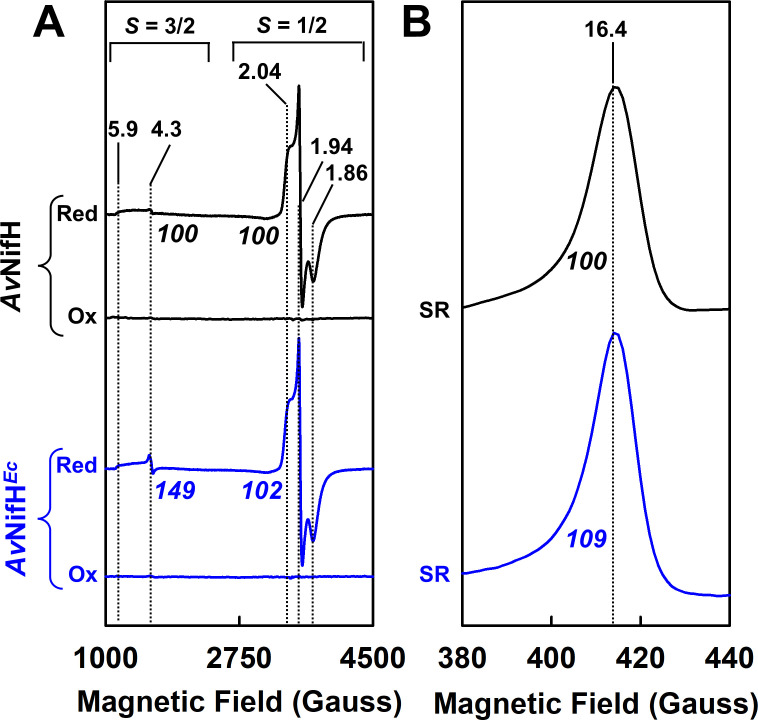
EPR analyses of *Av*NifH*^Ec^*. (A) Perpendicular- and (B) parallel-mode EPR spectra of indigo disulfonate-oxidized (Ox), dithionite-reduced (Red), and super-reduced (SR) forms of *Av*NifH*^Ec^* (blue) as compared to those of its native *Av*NifH counterpart (black). The signal intensities of the EPR spectra are normalized based on the Fe contents of *Av*NifH*^Ec^* (3.3 ± 0.2 mol Fe/mol protein) and *Av*NifH (3.9 ± 0.3 mol Fe/mol protein). The percentages of the signal intensities of *Av*NifH*^Ec^* as compared to those of *Av*NifH (set at 100%) are indicated below the EPR traces. Note the characteristic shallow, broad *S* = 3/2 signals observed in the spectra of both *Av*NifH and *Av*NifH*^Ec^* in the dithionite-reduced state, which are shown at 10-fold enhanced intensities in Fig. S1.

The [Fe_4_S_4_] cluster of *Av*NifH*^Ec^* has the same ability as that of its native *Av*NifH counterpart (1, 2, 11, 27) to adopt three oxidation states: the oxidized (+2) state, the reduced (+1) state, and the super-reduced, all-ferrous (0) state. Accordingly, like the native *Av*NifH, *Av*NifH*^Ec^* is EPR-silent in the oxidized state (Fig. 3A, “Ox”) but displays a mixture of *S* = 3/2 (*g* = 5.9, 4.3) and *S* = 1/2 (*g* = 2.04, 1.94, 1.86) perpendicular-mode EPR signals in the reduced state (Fig. 3A, “Red”) (11) as well as a *g* = 16.4 parallel-mode signal in the super-reduced, all-ferrous state (Fig. 3B, “SR”) (27). Other than a slightly stronger *g* = 4.31 feature of its *S* = 3/2 signal in the reduced state, the EPR features of *Av*NifH*^Ec^* are comparable in magnitude to those of its native *Av*NifH counterpart, with the intensities of the *S* = 3/2 (reduced), *S* = 1/2 (reduced), and *g* = 16.4 (super-reduced) signals of *Av*NifH*^Ec^* being 149%, 102%, and 109%, respectively, of those of the corresponding features of *Av*NifH (Fig. 3A and B).

**Fig 4:**
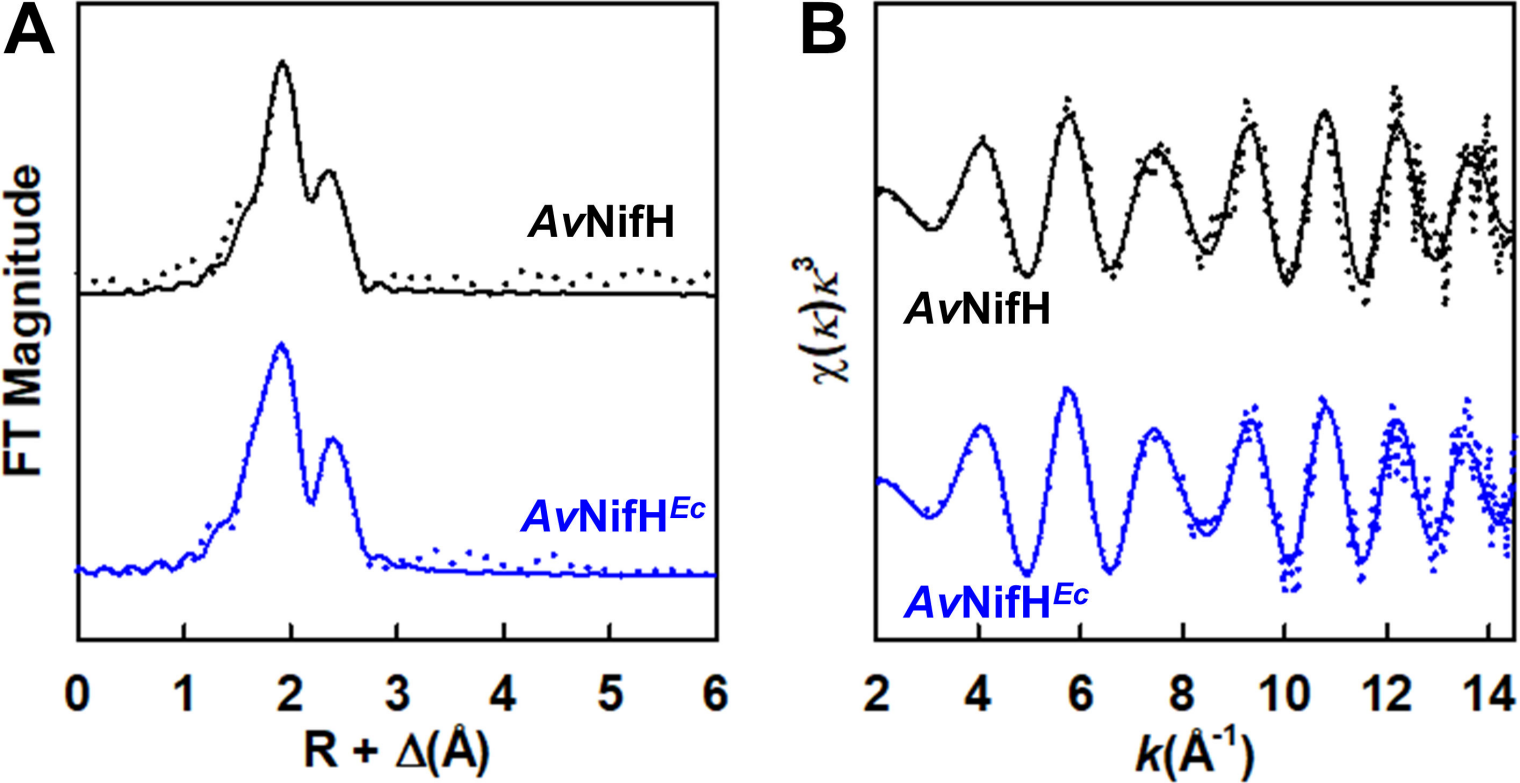
Fe K-edge XAS analysis of *Av*NifH*^Ec^*. Shown are the Fourier-transformed (A) and *k*^3^-weighted (B) EXAFS data (dotted) and best fits (solid) of *Av*NifH*^Ec^* (blue) as compared to those of its native *Av*NifH counterpart (black). See Fig. S2 for Fe K-edge absorption spectra, Table S2 for Fe K-edge energies, and Tables S3 and S4 for details of fits.

The close resemblance between the *Av*NifH*^Ec^*- and *Av*NifH-associated clusters is also reflected by a high degree of similarity between the Fe K-edge XAS/EXAFS data of reduced *Av*NifH*^Ec^* and *Av*NifH, both showing a primary component in the Fourier transform (FT) that requires Fe–S scattering pathways of ~2.3 Å, as well as a smaller secondary feature in the FT that requires two sets of Fe–Fe scatterers: one at 2.5 Å and the other at 2.7 Å (Fig. 4; Tables S2 to S4). There is a minor shift in the Fe K-edge energy of *Av*NifH*^Ec^* (7,118.3 eV) from that of *Av*NifH (7,117.9 eV) (Table S2); however, the overall similarity between the Fe K-edge XAS/EXAFS data of *Av*NifH*^Ec^* and *Av*NifH, along with the similarities among their metal contents, activities, and EPR features, suggests that the cluster environments in these proteins are highly similar.

**Fig 5:**
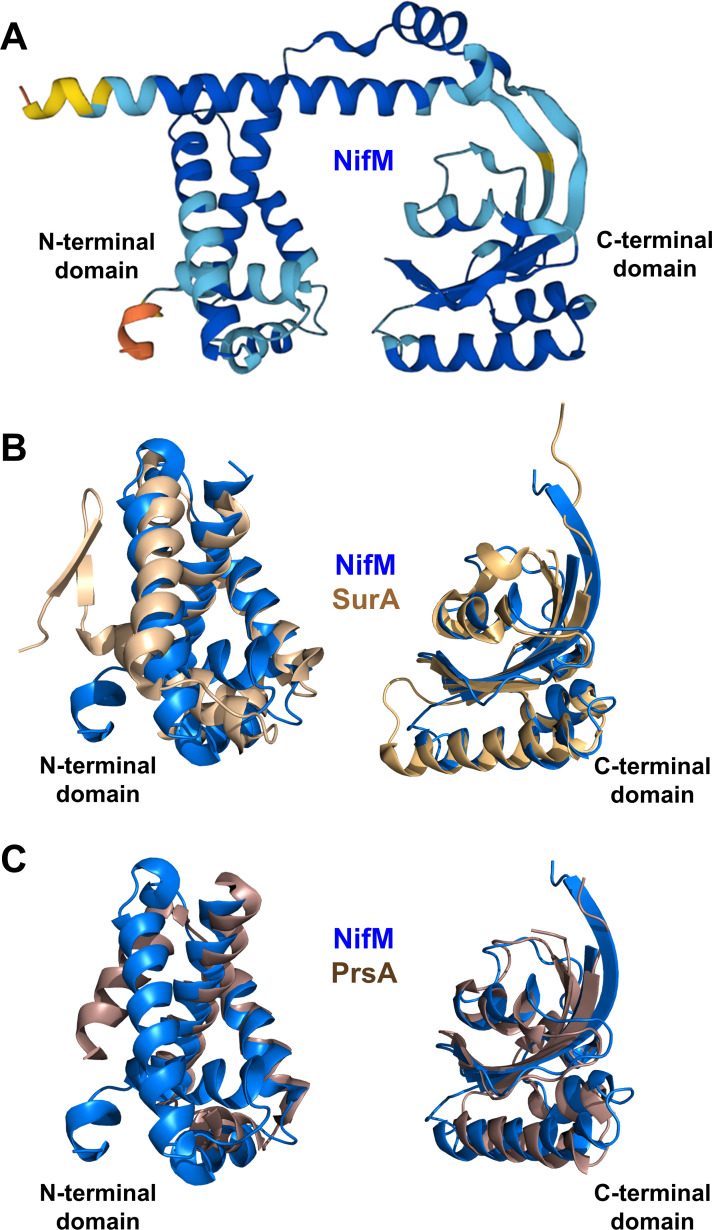
Structural comparison of *Av*NifM with SurA and PrsA. (A) AlphaFold structural prediction of *Av*NifM. Model confidence: blue, very high; cyan, confident; yellow, low; and orange, very low. (B and C) Overlay of the predicted N-terminal (left) and C-terminal (right) domains of *Av*NifM with those of (B) SurA (PDB: 1M5Y) or (C) PrsA (PDB: 6VJ4). The domains of NifM, SurA, and PrsA are colored blue, light brown, and dark brown, respectively.

The absolute requirement of *Av*NifM for the successful expression of *Av*NifH in *E. coli* points to a crucial role of *Av*NifM in facilitating the maturation of *Av*NifH into a functional protein. Previous sequence alignment and mutagenic analysis have led to the proposal that *Av*NifM is a peptidyl-prolyl isomerase (PPIase) involved in the transition between the *cis-* and *trans*-forms of the peptide bond of the conserved Pro258 residue in *Av*NifH (38). Yet, Pro258 is nearly 100% conserved in the NifH species from organisms with or without NifM, casting doubt on the proposed PPIase activity of NifM. Interestingly, based on the structural predictions available in the AlphaFold database (39, 40), *Av*NifM not only possesses a C-terminal domain with a PPIase fold but also has an N-terminal domain with striking resemblance to SurA and PrsA, two proteins known to facilitate protein folding (Fig. 5). A periplasmic chaperone found in many Gram-negative bacteria (e.g., *E. coli*), SurA possesses two PPIase domains but shows no *in vivo* PPIase activity (41); instead, it is known to facilitate the folding of outer membrane proteins in the periplasm (42, 43). Similarly, PrsA is an extracellular foldase that is best studied in certain Gram-positive bacteria (e.g., *Bacillus subtilis*) and, despite possessing the characteristic PPIase domain, it serves to assist the folding of a wide range of proteins, including pathogenicity factors and cell wall synthesis proteins (44–46). Given its architectural similarity to SurA and PrsA, it is likely that NifM also functions as a chaperone that is indispensable for the maturation of a soluble, cluster-replete form of NifH. The exact mode-of-action of NifM in this process, however, remains unclear.

**Fig 6:**
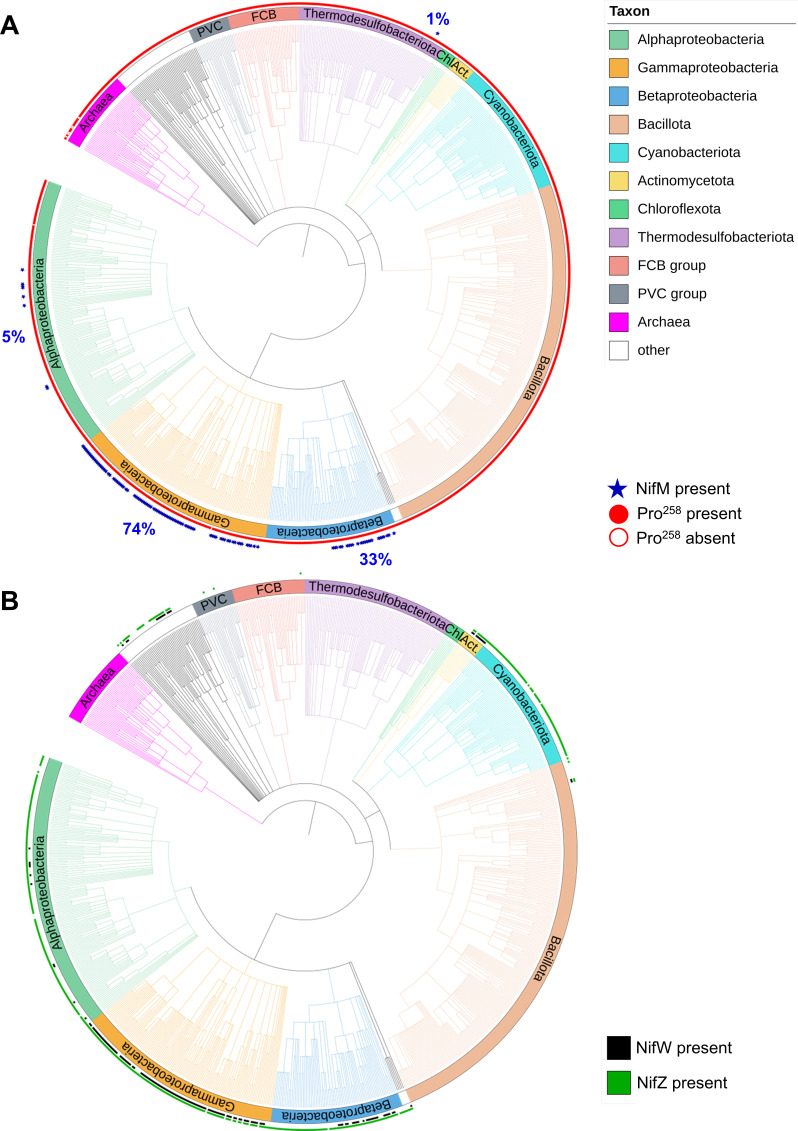
Phylogenetic tree of 943 organisms containing a total of 1,502 *nifH* homologs in their genomes. (A) Of these *nifH* containing organisms, 122 contain *nifM* sequences (indicated by blue stars). The percentages of *nifM* sequences found in 4 of the 11 identified taxonomic groups are shown in blue. The Pro258 residue of *Av*NifH, which has been proposed to be the substrate of NifM, is conserved in 99.5% of the NifH homologs (indicated by closed red circles). (B) Of these *nifH* containing organisms, 456 contain *nifZ* sequences (indicated by green squares). Analysis of the genomic neighborhoods of *nifZ* sequences has identified the presence of *nifW* sequences (indicated by black squares) in 159 organisms. Abbreviations: FCB_group, Fibrobacterota-Chlorobiota-Bacteroidota and PVC_group, Planctomycetota-Verrucomicrobiota-Chlamydiota.

It is interesting to note that while certain diazotrophs (e.g., *A. vinelandii*) require NifM for the maturation of NifH, most Nif (or homolog) possessing organisms do not contain NifM (Fig. 6A). The presence of NifM is largely limited to a subset of Proteobacteria (i.e., Gammaproteobacteria and Betaproteobacteria) and only occasionally observed in Alphaproteobacteria and other phyla (Fig. 6A). Despite the similarity of its C-terminal domain to PPIase, the N-terminal domain of NifM does not seem to be homologous to any known protein. As such, the evolutionary origin of this protein is unknown. Notably, the gene encoding NifM is almost always present alongside those encoding NifZ and NifW, two nitrogenase assembly/accessory proteins (Fig. 6B); moreover, the three *nif* genes are almost always grouped in the same genomic architecture: *nifWZM*. Given the participation of NifZ and NifW in the maturation of the catalytic NifDK component of *A. vinelandii* (47, 48), such a conserved genomic arrangement of *nifWZM* could imply a functional “grouping” of maturation proteins of nitrogenase and thereby offer additional support for the proposed role of NifM as a chaperone for the maturation of NifH. The question of why NifM is specifically required for the expression of certain NifH species, but not others, requires further investigation.

### Conclusion

In this work, we have successfully synthesized a fully active *Av*NifH with a high cluster occupancy upon co-expression of this protein with *Av*IscS/U and *Av*NifM in *E. coli*. Our biochemical and spectroscopic data confirm the structural and functional competence of this heterologously expressed *Av*NifH protein, and our phylogenetic analyses and structural predictions suggest a role of *Av*NifM as a chaperone for the maturation of a cluster-replete form of *Av*NifH. Given the crucial role of NifH in nitrogenase catalysis and assembly, this work supplies an essential component for piecing together a complete pathway for the heterologous expression of an active nitrogenase, an ongoing effort that involves further optimization of key parameters and, particularly, the FeS supplies in the expression system (49–53). Such an expression system of the two-component nitrogenase, along with that of its NifH component alone (as reported herein), could prove useful for the future development of biotechnological applications that harness the reducing prowess of nitrogenase for the production of valuable chemical commodities. Moreover, the expression system described in this work provides a useful tool for tackling the impact of maturation factors, such as *Av*NifM, in the synthesis of a functional *Av*NifH, as well as the relationship of various Nif proteins and homologs during evolution, which could prove instrumental in decoding the biosynthetic mechanism of nitrogenase while unraveling the evolutionary origins of this important metalloenzyme.

## MATERIALS AND METHODS

All the chemicals were purchased from Sigma-Aldrich (St. Louis, MO) and Thermo Fisher Scientific (Waltham, MA), except when noted otherwise. The experiments were performed either in a glove box or on a Schlenk line, under an atmosphere of Ar with an O_2_ concentration of <3 ppm.

### Strain construction

The genes encoding *A. vinelandii* NifH and NifM proteins were synthesized, codon-optimized for *E. coli* expression, and then cloned into pET-14b and pRSFDuet-1 vectors, respectively (GenScript, Piscataway, NJ). These constructs were then co-transformed along with a plasmid containing *iscSUA* and *hscABfdx* genes from *A. vinelandii*, which encodes a group of proteins responsible for FeS cluster assembly (34–37), into the *E. coli* strain BL21(DE3), resulting in a strain (YM565EE) that expresses His-tagged *Av*NifH (designated *Av*NifH*^Ec^*) when induced with isopropyl β-D-1-thiogalactopyranoside (IPTG). The plasmid carrying the *iscSUA* and *hscABfdx* genes was kindly provided by Prof. Silke Leimkhüler from the University of Potsdam, Germany.

### Cell growth and protein purification

In a BIOFLO 415 fermenter (New Brunswick Scientific) operated at 37°C with 200 rpm agitation and 10 L/min airflow, the *E. coli* strain YM565EE was cultivated in 10 L batches in LB medium (Difco) supplemented with 50 mM MOPS/NaOH (pH 7.4), 25 mM glucose, 2 mM ferric ammonium citrate, 19 mg/L kanamycin, 28 mg/L chloramphenicol, and 75 mg/L ampicillin. When optical density (OD_600_) reached 0.5, the airflow was terminated, and the fermenter was purged with N_2_ (ultra-high purity) at a rate of 1.5 L/min; additionally, the temperature was lowered to 24°C before 25 mM sodium fumarate, 2 mM cysteine was added to the culture, and the expression of His-tagged *Av*NifH*^Ec^* was induced upon addition of 250 µM IPTG. The protein was expressed for 16 h before the cells were harvested through centrifugation using a Thermo Fisher Scientific Legend XTR centrifuge. Subsequently, the protein was isolated using immobilized metal affinity chromatography using a method adapted from the purification of His-tagged nitrogenase proteins from *A. vinelandii* (54).

In a 200-L fermenter (New Brunswick Scientific) operated at 30°C with 100 rpm agitation and 30 L/min airflow, the *A. vinelandii* strains DJ1162, DJ1141, DJ1143, DJ1165, and DJ1041 expressing His-tagged *Av*NifH, *Av*NifDK, P-cluster precursor (i.e., P*-cluster, a [Fe_4_S_4_]-like cluster pair) containing *Av*NifDK, P-cluster containing *Av*NifDK, and M-cluster precursor (i.e., L-cluster, a [Fe_8_S_9_C] cluster) containing *Av*NifEN (47, 55), respectively, were grown in 180-L batches in Burke’s minimal medium supplemented with 2 mM ammonium acetate. Cell growth was monitored at OD_436_ using a Spectronic 20 Genesys spectrometer (Spectronic Instruments), and following the exhaustion of ammonia, the cells were de-repressed for 3 h before being harvested with a flow-through centrifugal harvester (Cepa). Published methods were used to purify His-tagged *Av*NifH, *Av*NifDK, and *Av*NifEN proteins (47, 55).

### Metal analysis

The Fe contents of *Av*NifH and *Av*NifH*^Ec^* were analyzed using inductively coupled plasma optical emission spectroscopy (ICP-OES) with an iCAP7000 ICP-OES instrument (Thermo Scientific). The calibration of the instrument was carried out using standard solutions made from dilution of a 1 mg/mL stock solution of elemental Fe (Thermo-Fisher Scientific). The analysis began by combining the protein sample with 100 µL of concentrated sulfuric acid (H_2_SO_4_) and 100 µL of concentrated nitric acid (HNO_3_), followed by heating at 250°C for 30 min. This process was repeated until the solution turned colorless. Subsequently, the solution was allowed to cool to room temperature, followed by dilution of the solution to a total volume of 7.5 mL with 2% HNO_3_ and determination of the metal content.

### Substrate reduction assays

C_2_H_2_- and N_2_-reduction assays were performed at 30°C in 9.5-mL vials fitted with rubber serum stoppers and metal caps (DWK Life Science, Millville, NJ). The C_2_H_2_-reduction assay contained an atmosphere of 0.1 atm C_2_H_2_ and 0.9 atm Ar, while the N_2_-reduction assay contained 1.0 atm of N_2_. Each reaction contained 25 mM Tris–HCl (pH 8.0), 2.5 mM ATP, 5.0 mM MgCl_2_, 30 mM creatine phosphate, 0.125 mg of creatine phosphokinase, and 20 mM sodium dithionite (Na_2_S_2_O_4_) in a total volume of 1 mL. The reaction was initiated by the addition of 2.4 mg of *Av*NifDK and 0.36 mg of *Av*NifH or *Av*NifH*^Ec^*, incubated at 30°C for 10 min, quenched with EDTA, and analyzed for product formation. To detect C_2_H_4_ as a product of C_2_H_2_-reduction, 250 µL of the headspace was injected into a GC-FID (SRI Instruments, Torrance, CA) equipped with a packed Poropak N column (Restek, Bellefonte, PA). Calibration was achieved by injecting 15 ppm C_2_H_4_ gas standard under the same conditions. To detect NH_3_ as the product of N_2_-reduction, 100 µL of the reaction was added to an o-phthalaldehyde (OPA) solution that contained, in a total volume of 1 mL, 10 mM OPA and 2.5 mM 2-mercaptomethanol in a 50 mM potassium phosphate buffer (pH 7.8). The mixture was allowed to sit at room temperature for 3 h, followed by measurement using a fluorescence spectrophotometer (RF-5301PC, Shimadzu Co., Ltd., Japan) using an excitation wavelength of 361 nm and an emission wavelength of 423 nm.

### P-cluster maturation assays

Each assay contained 25 mM Tris–HCl (pH 8.0), 0.45 mg of P-cluster precursor (i.e., P*-cluster; 2 × [Fe_4_S_4_]) containing, yet cofactor-deficient NifDK from *A. vinelandii nifH*-deletion strain DJ1165 (47), 0.5 mg of *Av*NifH or *Av*NifH*^Ec^*, 20 mM Na_2_S_2_O_4_, 0.8 mM ATP, 1.6 mM MgCl_2_, 10 mM creatine phosphate, 8 units of creatine phosphokinase, and 10 mL of isolated M-clusters (56) in a total volume of 0.9 mL. The reaction was incubated at 30°C for 60 min and subsequently split into triplicates in three 9.5-mL vials, each containing 1.05 mg of NifH, 25 mM Tris–HCl (pH 8.0), 2.5 mM ATP, 5.0 mM MgCl_2_, 30 mM creatine phosphate, 0.125 mg of creatine phosphokinase, and 20 mM Na_2_S_2_O_4_ in a total volume of 0.7 mL, and either C_2_H_2_ or N_2_ in the headspace as the substrate of the C_2_H_2_- or N_2_-reduction assay. The reaction mixture was then incubated at 30°C for 10 min and analyzed for product formation as described above.

### M-cluster maturation assays

Each assay contained 25 mM Tris–HCl (pH 8.0), 0.45 mg of P-cluster containing, yet cofactor-deficient NifDK from *A. vinelandii nifB*-deletion strain DJ1143 (47), 1.2 mg of *Av*NifH or *Av*NifH*^Ec^*, 1.0 mg M-cluster precursor (i.e., L-cluster [Fe_8_S_9_C]) containing NifEN from *A. vinelandii* strain DJ1041 (55), 0.4 mM homocitrate, 0.4 mM Na_2_MoO_4_, 2.4 mM ATP, 4.8 mM MgCl_2_, 30 mM creatine phosphate, 24 units of creatine phosphokinase, and 20 mM Na_2_S_2_O_4_ in a total volume of 0.9 mL. The reaction was incubated at 30°C for 60 min and subsequently split into triplicates in three 9.5-mL vials, each containing 1.05 mg of NifH, 25 mM Tris–HCl (pH 8.0), 2.5 mM ATP, 5.0 mM MgCl_2_, 30 mM creatine phosphate, 0.125 mg of creatine phosphokinase, and 20 mM Na_2_S_2_O_4_ in a total volume of 0.7 mL, and either C_2_H_2_ or N_2_ in the headspace as the substrate of C_2_H_2_- or N_2_-reduction assay. The reaction mixture was then incubated at 30°C for 10 min and analyzed for product formation as described above.

### EPR analysis

EPR samples were assembled and flash frozen in liquid nitrogenase in a glove box (Vacuum Atmospheres) filled with Ar and maintained at an O_2_ concentration of <3 ppm. Three types of samples were prepared for EPR analysis as follows: (i) reduced (Red) samples, which contained 10% (vol/vol) glycerol, 250 mM imidazole, 2 mM Na_2_S_2_O_4_, and 25 mM Tris–HCl (pH 8.0); (ii) oxidized (Ox) samples, which were prepared by incubating the reduced samples with excess indigo disulfonate for 5 min; and (iii) the super-reduced (SR) samples, which were prepared by adding excess europium (II) ethylene glycol-bis(β-aminoethyl ether)-*N*,*N*,*N*′,*N*′-tetraacetic acid (Eu-EGTA), followed by removal of Eu-EGTA on a G25 desalting column (Sigma-Aldrich).

EPR spectra were recorded by an ESP 300E spectrometer (Bruker) interfaced with an ESR-9002 liquid-helium continuous-flow cryostat (Oxford Instruments). The measurements were taken using a microwave power of 50 mW, a gain factor of 5 × 10^4^, a modulation frequency of 100 kHz, and a modulation amplitude of 5 G. Five scans of perpendicular-mode EPR spectra were recorded for each sample at 10 K (reduced samples) or 15 K (oxidized samples) using a microwave frequency of 9.62 GHz; and eight scans of parallel-mode EPR spectra were recorded for each sample at 10 K (super reduced samples) using a microwave frequency of 9.38 GHz.

### XAS spectra

Fe K-edge, XAS spectra were collected with a SPEAR3 storage ring current of ~500 mA and an energy level of 3.0 GeV at two SSRL (Stanford Synchrotron Radiation Lightsource) beamlines: beamline 7-3, which uses a 30-element solid-state Ge detector (Canberra) and beamline 9-3, which uses a 100-element Ge monolith solid-state detector (Canberra). For the Fe scans, an Fe foil was positioned in the beam path before the ionization chamber (I_0_) and scanned simultaneously for energy calibration, with the first inflection point of the edge assigned to 7,112.0 eV. The potential occurrence of photoreduction was closely monitored by scanning the same spot on the sample twice, which allowed for a comparison between the first derivative peaks associated with the edge energy during the data acquisition process.

The detector channels from the scans were inspected, calibrated, and averaged using the software EXAFSPAK (23). Subsequently, the data were processed for EXAFS analysis with PYSPLINE (57) to obtain *χ*(k). PYSPLINE was employed to subtract a second-order background across the entire data range and to create a spline function that modeled the background absorption throughout the EXAFS region. A four-region spline was selected, utilizing polynomials of orders 2, 3, and 3 over the post-edge region, and the data were normalized to obtain an edge jump of 1.0 at 7,130 eV. For a specific absorber-scatterer pair, theoretical phase and amplitude parameters were computed using FEFF 8.40 (58). Parameters for each species were calculated based on a suitable model derived from the crystal structure of the [Fe_4_S_4_] cluster in NifH (PDB code 1G5P) (59).

In every analysis conducted, the coordination number of a specific shell (*N*) was kept as a fixed parameter and changed iteratively in integer steps, while the bond lengths (*R*) and mean-square deviation (*σ*²) were permitted to vary without restriction. The estimated uncertainties in *R*, *σ*², and *N* are 0.02 Å, 0.1 × 10⁻³ Å², and 20%, respectively. For the Fe K-edge data, the amplitude reduction factor *S*_₀_ was set at 1.0, while the edge-shift parameter Δ*E*_₀_ was allowed to vary as a singular value across all shells. Therefore, in any particular fit, the number of variable parameters typically equaled two times the number of shells plus 1. The goodness of fit parameters were determined in accordance with the following formulae:


$$F=\sqrt{\sum k^{6}{\left(\chi_{{}_{exp}}-\chi_{{}_{calc}}\right)}^{2}},$$

$$F^{\prime }=\sqrt{\sum k^{6}{\left(\chi_{{}_{exp}}-\chi_{{}_{calc}}\right)}^{2}/\sum k^{6}\chi_{{}_{exp}}{}^{2}}.$$

Analysis of the pre-edge was carried out on the Fe K-edge fluorescence data, which was normalized to achieve an edge jump of 1.0 at 7,130 eV in PYSPLINE. The features of the pre-edge were fit within the energy range of 7,108 to 7,117 eV using methods detailed elsewhere (60, 61). The fitting was conducted using the Fityk program (62), with pseudo-Voigt functions comprising a mixture of Gaussian and Lorentzian functions at a 50:50 ratio.

### AlphaFold models

The structural information of *Av*NifM was retrieved from the AlphaFold Protein Structure Database for entry P14890 (39, 63). The predicted structure of the N-terminal domain of *Av*NifM was then submitted to NCBI VAST (64), which resulted in a hit for the crystal structure of a truncated SurA (PDB code 2PV3) (65) and, consequently, a comparative structural analysis between *Av*NifM and the full-length SurA structure (PDB code 1M5Y) (66). Further analysis of the predicted structure of *Av*NifM using FoldSeek (67) led to the identification of PrsA (PDB code 6VJ4) as another close structural homolog of *Av*NifM (68). PyMOL was used to facilitate structural analysis and visualization (69).

### Phylogenetic analysis

The NifH sequences were retrieved from InterPro family IPR005977 (containing *Av*NifH) (70) and filtered through Reference Proteomes from UniProt (71) to decrease the total number of sequences, which resulted in the identification of 1,502 NifH homolog sequences from 943 organisms. The phylogenetic tree of the organisms was generated based on NCBI taxonomy with phyloT (database version 2022.3) (72) and visualized and annotated in iTOL version 6.7.3 (73). To investigate the presence or the absence of the residue corresponding to Pro258 of *Av*NifH, the NifH sequences were aligned with MAFFT (74) using automatic settings, and the alignment was trimmed with trimAl (75). The presence or the absence of NifM was investigated by retrieving NifM sequences from InterPro family IPR014282 (containing *Av*NifM) and matching the sequences by the taxon IDs of the organisms. Similarly, the presence or the absence of NifZ was investigated by retrieving NifZ sequences from InterPro family IPR007415 (containing *Av*NifZ). The identified NifZ sequences were then submitted to EFI-Genome Neighborhood Tool (76, 77), and the NifW sequences neighboring NifZ were annotated accordingly. In cases where more than one NifZ homolog was present in a given organism, the organism was annotated as containing NifW if at least one of the NifZ homologs had a neighboring NifW sequence.

## Data Availability

The data that support the findings of this study are available either within this article and its supplemental material files or upon reasonable request to the corresponding authors.

## SUPPLEMENTAL MATERIAL

Fig. S1 mbio.02572-23-s0001.pdf Perpendicular-mode EPR spectra.

Fig. S2 mbio.02572-23-s0002.pdf Fe K-edge absorption spectra.

Table S1 mbio.02572-23-s0003.pdf Specific activities.

Table S2 mbio.02572-23-s0004.pdf Fe K-edge energies.

Table S3 mbio.02572-23-s0005.pdf Fit parameters for the Fe K-edge EXAFS data of AvNifH.

Table S4 mbio.02572-23-s0006.pdf Fit parameters for the Fe K-edge EXAFS data of AvNifH-Ec.

ASM does not own the copyrights to Supplemental Material that may be linked to, or accessed through, an article. The authors have granted ASM a non-exclusive, world-wide license to publish the Supplemental Material files. Please contact the corresponding author directly for reuse.
