# Supplementary material for: Heterologous expression of a fully active Azotobacter vinelandii nitrogenase Fe protein in Escherichia coli
Source: mBio. 2023 Nov 1;14(6):e02572-23. doi: 10.1128/mbio.02572-23 (PMC10746259; doi:10.1128/mbio.02572-23)
Supplement: Fig. S1 — Perpendicular-mode EPR spectra. [file mbio.02572-23-s0001.pdf]

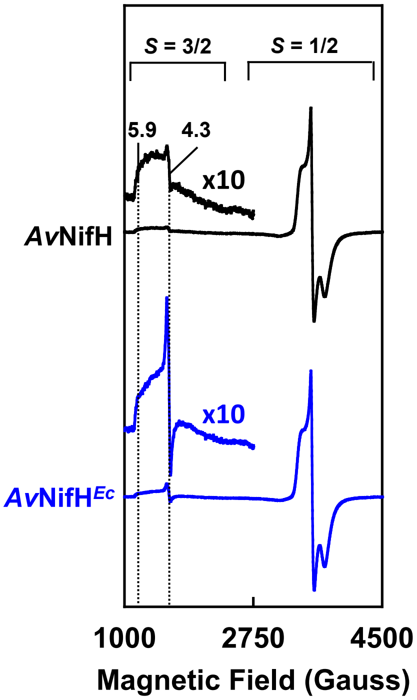

FIG S1. Perpendicular-mode EPR spectra of dithionite-reduced *Av*NifH^{Ec} and *Av*NifH (also see Fig. 3A for other relevant EPR spectra) with the insets showing the *S*=3/2 signals of the two proteins with the same *g* values (*g*=5.9, 4.3) at 10-fold enhanced intensities.
