# Supplementary figures and images for: Heterologous expression of a fully active Azotobacter vinelandii nitrogenase Fe protein in Escherichia coli
Source: mBio. 2023 Nov 1;14(6):e02572-23. doi: 10.1128/mbio.02572-23 (PMC10746259; doi:10.1128/mbio.02572-23)

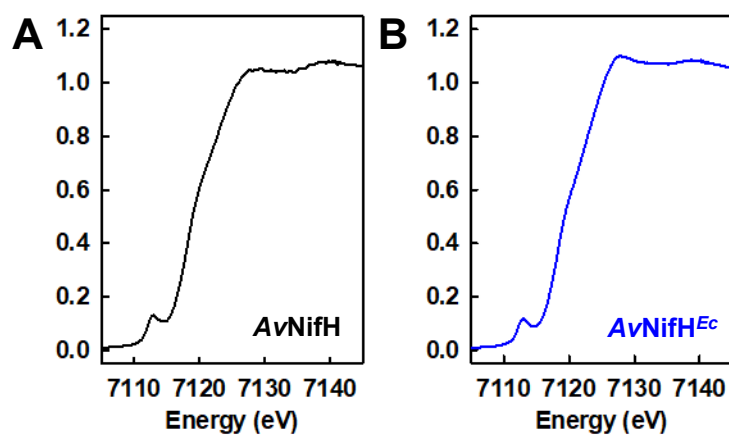

**FIG S2.** Fe K-edge absorption spectra of AvNifH and AvNifH<sup>Ec</sup>.

Supplement: Fig. S2 — Fe K-edge absorption spectra. [file mbio.02572-23-s0002.pdf]
