# Supplementary material for: Heterologous expression of a fully active Azotobacter vinelandii nitrogenase Fe protein in Escherichia coli
Source: mBio. 2023 Nov 1;14(6):e02572-23. doi: 10.1128/mbio.02572-23 (PMC10746259; doi:10.1128/mbio.02572-23)
Supplement: Table S1 — Specific activities. [file mbio.02572-23-s0003.pdf]

Table S1. Specific activities and Fe content of *Av*NifH and *Av*NifH^{Ec}.

**Activities**	***Av*NifH**			***Av*NifH^{Ec}**		
	nmol product/ mg protein/min *normalized based on Fe content*	*%*	*nmol product/ mg protein/min* *normalized based on 4 Fe/protein*	*nmol product/ mg protein/min* *normalized based on Fe content*	*%*	*nmol product/ mg protein/min* *normalized based on 4 Fe/protein*
Catalysis (as the reductase for NifDK as partner)						
C_2H_2-reduction (to C_2H_4)	545 $\pm$ 30	100 $\pm$ 6	2180 + 120	522 $\pm$ 40	96 $\pm$ 7	2088 $\pm$ 160
N_2-reduction (to NH_3)	320 $\pm$ 27	100 $\pm$ 8	1280 $\pm$ 108	248 $\pm$ 43	77 $\pm$ 13	992 $\pm$ 172
M-cluster maturation						
C_2H_2-reduction (to C_2H_4)	109 $\pm$ 10	100 $\pm$ 9	436 + 40	108 $\pm$ 9	99 $\pm$ 8	432 $\pm$ 36
P-cluster maturation						
C_2H_2-reduction (to C_2H_4)	88 $\pm$ 20	100 $\pm$ 23	352 + 80	92 $\pm$ 12	104 $\pm$ 14	368 $\pm$ 48
	nmol reduced C/ mmol protein *normalized based on Fe content*	*%*	*nmol reduced C/ mmol protein* *normalized based on 4 Fe/protein*	*nmol reduced C/ mmol protein* *normalized based on Fe content*	*%*	*nmol reduced C/ mmol protein* *normalized based on 4 Fe/protein*
Catalysis (as an independent reductase) CO_2-reduction (to CO)	7.8 $\pm$ 0.2	100 $\pm$ 3	31.2 + 0.8	7.3 + 0.1	94 $\pm$ 1	29.2 + 0.4
**Fe content**	*mol Fe/ mol protein*	*%*		*mol Fe/ mol protein*	*%*	
	3.9 $\pm$ 0.3	100 $\pm$ 8		3.3 $\pm$ 0.2	85 $\pm$ 5	
