# Supplementary material for: Heterologous expression of a fully active Azotobacter vinelandii nitrogenase Fe protein in Escherichia coli
Source: mBio. 2023 Nov 1;14(6):e02572-23. doi: 10.1128/mbio.02572-23 (PMC10746259; doi:10.1128/mbio.02572-23)
Supplement: Table S2 — Fe K-edge energies. [file mbio.02572-23-s0004.pdf]

**Table S2.** Fe K-edge energies of proteins used in this study.

| Protein | Fe K-edge Energy (eV) |
|---|---|
| *Av*NifH | 7117.9 |
| *Av*NifH$^{Ec}$ | 7118.3 |
