# Supplementary material for: Heterologous expression of a fully active Azotobacter vinelandii nitrogenase Fe protein in Escherichia coli
Source: mBio. 2023 Nov 1;14(6):e02572-23. doi: 10.1128/mbio.02572-23 (PMC10746259; doi:10.1128/mbio.02572-23)
Supplement: Table S3 — Fit parameters for the Fe K-edge EXAFS data of AvNifH. [file mbio.02572-23-s0005.pdf]

Table S3. Fit parameters for the Fe K-edge EXAFS data of *Av*NifH between k = 2-14.5 $Å^{-1}$.

	Fe–S			Fe•••Fe				GOF	
**Fit**	**N**	**R(Å)**	**$\sigma^2(10^{-3})$**	**N**	**R(Å)**	**$\sigma^2(10^{-3})$**	**ΔE_0**	**F**	**F'**
1	1	2.33	-0.17				-2.18	1670	617
2	2	2.33	0.59				-2.10	1264	537
3	3	2.32	2.35				-3.06	1247	533
4	4	2.32	3.94				-3.52	1411	567
5	3	2.32	2.50	1	2.75	2.30	-2.59	739	410
6	3	2.33	2.33	2	2.76	5.92	-1.50	790	424
7	3	2.33	2.24	3	2.76	9.04	-0.99	881	448
8	3	2.32	3.45	2	2.73	5.45	-4.57	715	404
				1	2.50	4.57			
