# Supplementary material for: Heterologous expression of a fully active Azotobacter vinelandii nitrogenase Fe protein in Escherichia coli
Source: mBio. 2023 Nov 1;14(6):e02572-23. doi: 10.1128/mbio.02572-23 (PMC10746259; doi:10.1128/mbio.02572-23)
Supplement: Table S4 — Fit parameters for the Fe K-edge EXAFS data of AvNifH-Ec. [file mbio.02572-23-s0006.pdf]

Table S4. Fit parameters for the Fe K-edge EXAFS data of *Av*NifH^{Ec} between k = 2-14.5 Å^{-1}.

	Fe–S			Fe•••Fe				GOF	
**Fit**	**N**	**R(Å)**	**$\sigma^2(10^{-3})$**	**N**	**R(Å)**	**$\sigma^2(10^{-3})$**	**ΔE_0**	**F**	**F'**
1	1	2.33	-0.09				-2.51	1899	679
2	2	2.33	1.31				-2.81	1372	578
3	3	2.32	3.07				-4.01	1208	542
4	4	2.32	4.64				-4.91	1226	546
5	3	2.32	3.31	1	2.75	1.43	-2.79	574	373
6	3	2.33	3.05	2	2.75	5.16	-1.68	680	407
7	4	2.33	4.54	2	2.75	4.74	-2.33	685	408
8	4	2.32	4.83	1	2.75	1.19	-3.58	540	362
9	4	2.31	5.56	1	2.74	0.68	-6.01	494	347
				1	2.53	8.59			
10	3	2.30	3.73	1	2.73	0.11	-7.64	486	343
				1	2.56	4.70			
11	3	2.29	4.20	2	2.71	3.26	-8.19	471	338
				1	2.53	1.66			
